# Reduction of double-strand DNA break repair exacerbates vascular aging

**DOI:** 10.18632/aging.205066

**Published:** 2023-10-02

**Authors:** Samuel I. Bloom, Jordan R. Tucker, Daniel R. Machin, Hossein Abdeahad, AdeLola O. Adeyemo, Tyler G. Thomas, R. Colton Bramwell, Lisa A. Lesniewski, Anthony J. Donato

**Affiliations:** 1 Department of Nutrition and Integrative Physiology, University of Utah, Salt Lake City, UT 84148, USA; 2 Department of Internal Medicine, Division of Geriatrics, University of Utah School of Medicine, Salt Lake City, UT 84148, USA; 3 Department of Nutrition and Integrative Physiology, Florida State University, Tallahassee, FL 32304, USA; 4 Geriatric Research, Education and Clinical Center, Veteran’s Affairs Medical Center-Salt Lake City, Salt Lake City, UT 84148, USA; 5 Nora Eccles Harrison Cardiovascular Research and Training Institute, The University of Utah, Salt Lake City, UT 84148, USA; 6 Department of Biochemistry, University of Utah, Salt Lake City, UT 84148, USA

**Keywords:** aging, DNA damage, vascular function, endothelial cell, senescence, oxidative stress, arterial stiffness

## Abstract

Advanced age is the greatest risk factor for cardiovascular disease (CVD), the leading cause of death. Arterial function is impaired in advanced age which contributes to the development of CVD. One underexplored hypothesis is that DNA damage within arteries leads to this dysfunction, yet evidence demonstrating the incidence and physiological consequences of DNA damage in arteries, and in particular, in the microvasculature, in advanced age is limited. In the present study, we began by assessing the abundance of DNA damage in human and mouse lung microvascular endothelial cells and found that aging increases the percentage of cells with DNA damage. To explore the physiological consequences of increases in arterial DNA damage, we evaluated measures of endothelial function, microvascular and glycocalyx properties, and arterial stiffness in mice that were lacking or heterozygous for the double-strand DNA break repair protein ATM kinase. Surprisingly, in young mice, vascular function remained unchanged which led us to rationalize that perhaps aging is required to accumulate DNA damage. Indeed, in comparison to wild type littermate controls, mice heterozygous for ATM that were aged to ~18 mo (Old ATM +/−) displayed an accelerated vascular aging phenotype characterized by increases in arterial DNA damage, senescence signaling, and impairments in endothelium-dependent dilation due to elevated oxidative stress. Furthermore, old ATM +/− mice had reduced microvascular density and glycocalyx thickness as well as increased arterial stiffness. Collectively, these data demonstrate that DNA damage that accumulates in arteries in advanced age contributes to arterial dysfunction that is known to drive CVD.

## INTRODUCTION

Advanced age is the greatest risk factor for the development of cardiovascular disease (CVD), which is the leading cause of death [1, 2]. Independent of overt CVD and other risk factors, advanced age results in impaired arterial and microvascular function, characterized by reductions in endothelium-dependent dilation (EDD) and microvascular health. These microvascular alterations with advanced age include reductions in microvascular density and increases in penetrability of the endothelial glycocalyx, a dynamic gel-like structure that lines endothelial cells and regulates redox balance, permeability, and responses to shear stress [3, 4]. Additionally, with advancing age large artery stiffness increases, as measured by arterial pulse wave velocity (PWV) [5, 6]. Importantly, age-related changes in EDD, microvascular health, and PWV precede and predict CVD diagnosis, morbidity, and mortality [5–8]. Despite compelling evidence that advanced age results in arterial dysfunction that leads to CVD, the mechanisms responsible for this dysfunction remain incompletely understood. It has been proposed that damage to the DNA of vascular cells is a key player in the development of age-related arterial dysfunction and CVD [9, 10]; however, there is a paucity of data demonstrating the abundance of arterial DNA damage in advanced age and the physiological consequences thereof.

With advancing age, cells accumulate molecular damage that can impair biochemical and thus cellular function [11]. However, within cells, most structures can be replaced or recycled as long as DNA, the code for those cellular components remains intact [12]. Conversely, the DNA damage theory of aging posits that damage to DNA has a sustained impact because it adversely affects DNA replication and transcriptional machinery or results in mutations that permanently alter cellular function [12]. Furthermore, because mutagenesis is a primary driver of cancer, cells are endowed with a robust DNA damage-response machinery. This includes ataxia-telangiectasia mutated (ATM), a Ser/Thr kinase that orchestrates the cellular response to double-strand breaks [13]. Following, DNA damage, ATM phosphorylates and activates tumor suppressor pathways [12]. Consequently, to prevent cells with damaged DNA from dividing, DNA damage and activation of ATM act as a potent effector of cell fate decisions including apoptosis or cellular senescence [12].

Arterial cells are some of the slowest cycling cells [14], the first to become senescent [15], and are highly susceptible to apoptosis [16]. Based on this understanding, DNA damage in the vasculature would be particularly deleterious because it impacts cells that may persist in a dysfunctional state for a long time or, if the damage is severe enough, leads to cell fate choices associated with age-related dysfunction [17, 18]. Despite this, several important and unanswered questions remain. For example, evidence of DNA damage in arterial cells with healthy aging is sparse [17]. Furthermore, the impact of DNA damage, and in particular, double-strand DNA breaks which are the most severe form of DNA damage, on arterial function in the context of aging remains incompletely understood. Accordingly, the purpose of the present study was to provide evidence of the abundance of DNA damage in arterial cells with advancing age and to explore the physiological consequences of an increased burden of double-strand DNA breaks due to reductions in repair capacity.

## METHODS

### Ethical approval and animals

All animal studies were performed in compliance with the Guide and Use of Laboratory Animals and were approved by the University of Utah and the Veteran’s Affairs Medical Center – Salt Lake City (VAMC-SLC). ATM^tm1AWB^ mice on a *129S6/SvEvTac* genetic background were used. ATM +/+, ATM +/−, and ATM −/− mice were housed in an animal care facility at the University of Utah and C57BL/6 mice were housed at the VAMC-SLC. All mice were housed on a 12:12 light dark cycle and fed normal rodent chow (Teklad Global soy protein-free extruded rodent diet, 2920X) and water ad libitum. Mouse characteristics are displayed in Table 1. Young (Yg) ATM mice were studied at ~5 mo of age. Old ATM mice were studied at ~18 mo. Male and female mice were used in approximately equal distributions (Table 1). Studies were not powered to detect sex differences, however, differences between sexes were not detected. For all outcomes evaluated in mice, individual data points with a black border denote female mice, whereas individual data points matching group colors denote male mice. For assessments of DNA damage in lung endothelial cells, young 2.7 ± 0.0 mo C57BL/6 male mice were obtained from Charles River Inc. Old 27.3 ± 0. 2 mo C57Bl/6 mice were obtained from the National Institute of Aging colony maintained by Charles River Inc.

**Table 1:** Mouse characteristics.

	**Yg ATM +/+**	**Yg ATM +/−**	**Yg ATM −/−**	**Old ATM +/+**	**Old ATM +/−**
Age (months)	4.9 ± 0.2	4.9 ± 0.2	5.0 ± 0.2	18.1 ± 0.1	18.4 ± 0.1
N	17	22	15	21	22
Number male (#)	11	10	7	15	10
Body mass (g)	30.4 ± 1.5	26.4 ± 0.8	27.2 ± 1.5	48.3 ± 2.9^*#†^	43.5 ± 3.1^*#†^
Heart mass (mg)	146.8 ± 5.9	132.5 ± 4.6	118.6 ± 4.4	201.8 ± 11.7^*#†^	196.7 ± 10.3^*#†^
Heart mass: body mass (mg/g)	5.0 ± 0.3	5.2 ± 0.2	4.4 ± 0.2	4.5 ± 0.6	4.9 ± 0.4
Quadriceps mass (mg)	369.4 ± 22.8	383.5 ± 17.9	287.5 ± 18.4^*#^	396.0 ± 16.3^*†^	322.2 ± 11.2^*#‡^
Quadriceps mass: body mass (mg/g)	12.7 ± 1.0	15.4 ± 0.5^*^	11.3 ± 1.1^#^	8.2 ± 0.5^#^	7.9 ± 0.6^#^
Spleen mass (mg)	99.1 ± 3.9	102.5 ± 6.1	104.8 ± 23.9	166.8 ± 49.0	212.1 ± 33.7^*#†‡^
Spleen mass: body mass (mg/g)	3.3 ± 0.2	4.1 ± 0.3	4.6 ± 1.5	4.3 ± 1.7	5.4 ± 1.0
PG adipose mass (g)	0.9 ± 0.2	0.7 ± 0.1	1.2 ± 0.2	2.2 ± 0.2^*#†^	2.3 ± 0.3^*#†^
PG adipose mass: body mass (mg/g)	25.2 ± 4.1	25.2 ± 2.8	38.2 ± 3.6^*^	47.5 ± 1.7^*#^	52.3 ± 4.4^*#†^
Kidney mass (mg)	365.8 ± 30.3	343.2 ± 17.6	323.6 ± 16.8	544.2 ± 22.3^*#†^	501.4 ± 18.6^*#†^
Kidney mass: body mass (mg/g)	12.1 ± 1.0	13.1 ± 0.4	11.7 ± 0.5	11.0 ± 0.5	12.3 ± 0.8
Liver mass (g)	1.6 ± 0.1	1.3 ± 0.1	1.7 ± 0.1	2.8 ± 0.3^*#†^	2.3 ± 0.2^*#†‡^
Liver mass: body mass (mg/g)	54.0 ± 3.2	50.5 ± 3.2	58.2 ± 5.5	60.1 ± 4.3	53.0 ± 1.9

Data are mean ± SEM; Abbreviations: Yg: young; PG: perigonadal. ^*^p < 0.05 vs. Yg ATM +/+, ^#^*p* < 0.05 vs. Yg ATM +/−, ^†^*p* < 0.05 vs. Yg ATM −/−, ^‡^*p* < 0.05 vs. Old ATM +/+.

### Primary lung endothelial cell isolation and culture

Young and old C57Bl/6 mice were euthanized by exsanguination while under isoflurane anesthesia, perfused with 0.9% saline through the right ventricle until effluent ran clear, and lungs from 3 mice from the same age group were dissected and placed into cold isolation media containing DMEM, 20% FBS, and 1% penicillin-streptomycin. Lungs were then mechanically digested using sterile scissors and placed into pre-warmed digestion media containing DMEM and 2 mg/mL collagenase type 2, and then incubated at 37ºC and gently agitated for 45 min. Lungs were further mechanically digested by trituration with a 14-gauge 6” metal cannula attached to a 30 mL syringe. Single cell suspensions were dispensed through a 70 μm disposable cell strainer (Falcon #352350) into a 50 mL conical tube. The sieve was washed with 10 mL of isolation medium to stop digestion, and single cell suspensions were spun at 1,000 rpm for 10 min at 4ºC. The supernatant was aspirated and cells were resuspended in 2 mL 0.1% BSA in 1× PBS. Cells were then tumbled with anti-PECAM (CD-31) beads bound to Sheep anti-rat IgG Dynabeads for 30 minutes and cells were isolated using a magnetic separator. Cells were plated onto fibronectin-coated glass coverslips and grown until 40–60% confluence. Cells were fixed in 4% paraformaldehyde pH 7.4 for 15 min at room temperature and then stored in 1× PBS at 4ºC overnight prior to immunofluorescence.

Human lung microvascular endothelial cells were purchased from Lonza from two young (29 ± 1 year old, female, one Black non-smoker, one Hispanic smoker) and two old (67 ± 1 year old, female, one Caucasian non-smoker, one Hispanic smoker) donors. Cells were plated onto fibronectin-coated glass coverslips and grown until 40–60% confluence, fixed in 4% paraformaldehyde pH 7.4 for 15 min at room temperature, and then stored in 1× PBS at 4ºC overnight prior to immunofluorescence.

### Immunofluorescence, imaging, and analysis

For primary lung endothelial cells, glass coverslips containing endothelial cells were placed in 100% methanol at −20ºC for 15 min, and then rehydrated in 1× PBS for 5 min at room temperature. Cells were placed in a blocking solution containing 1 mg/mL BSA, 3% goat serum, 0.1% Triton-X100, and 1 mM EDTA, all in 1× PBS for 30 min at room temperature. Cells were then incubated in 1:500 53BP1 (Novus Biologicals, NB100-0304, Rabbit) antibody in blocking solution for 1 hour at room temperature. Samples were washed 3 × 5 min in 1× PBS, followed by incubation in 1:500 Alexa Fluor 555 (Invitrogen, A-21429, Goat anti-Rabbit) in blocking solution for 1 hour at room temperature. Samples were washed 3 times in 1× PBS for 5 min and then twice in 2× saline sodium citrate (SSC) containing 0.1% Tween 20 for 10 min at 60ºC, followed by washing in 2× SSC and 1× SSC and diH_2_O for 10 min each all at room temperature. Samples were mounted DAPI Fluoromount G (VWR – Catalog #102092-102), coverslipped, weighted with 1 kg weight for 5 min, and stored in a dark container until imaging. Cells were imaged on an Olympus Fluoview FV1000 confocal microscope at 100× magnification. All samples were imaged using the same acquisition settings. 1 μm Z-slices through the entirety of the nucleus were imaged and compiled using Fiji Is Image J. Discrete 53BP1 foci were quantified from 335 cells from young mice, averaging 42 cells per experiment, and from 201 cells from old mice, averaging 40 cells per experiment. For analysis of mouse cells, individual data points are displayed with a border matching group colors to denote that cells were derived from male mice. Discrete 53BP1 foci were quantified from 64 cells from young humans averaging, 16 cells per experiment, and 57 from old humans, averaging 19 cells per experiment. For analysis of human cells, individual data points are displayed with a black border to denote that all cells were derived from female donors.

For assessment of DNA damage in arterial samples from mice, the abdominal aorta was collected following euthanasia, cleaned of perivascular adipose tissue, placed in optimal cutting medium, and frozen in cold 2-methylbutane before being placed at −80ºC. For aortic sections, 8 μm sections were cut and fixed for 15 min in 4% paraformaldehyde pH 7.4 at room temperature. The same reagents described above were used unless otherwise noted. Following fixation, samples were rinsed in 1× PBS for 5 min, and then placed in blocking solution for 15 min at room temperature. Samples were incubated in 1:500 53BP1 antibody, washed 3 × 5 min in 1× PBS and gently agitated, and then placed in 1:500 Alexa Fluor 555 in blocking solution for 1 hour at room temperature. Samples were washed 3 × 5 min in 1× PBS and gently agitated, and then mounted with DAPI, cover slipped, weighted with 1 kg weight for 5 min, and stored in a dark container until imaging. Aortic sections were imaged on an Olympus Fluoview FV1000 confocal microscope at 10× magnification using the mosaic feature to image the entirety of each aortic ring section. 1 μm Z-slices were imaged through the entirety of each aortic section and compiled using Fiji Is Image J. Discrete 53BP1 foci were quantified for 3128 cells from old ATM +/+ mice, averaging 782 cells per mouse, and 4280 cells from old ATM +/− mice, averaging 713 cells per mouse.

### Mesenteric artery endothelium-dependent and independent vasodilation

Mesenteric artery endothelium-dependent and -independent dilation were evaluated as described previously [19, 20]. Briefly, mice were euthanized by exsanguination under 2% isoflurane via cardiac puncture. Mesenteric arcades were removed and placed in cold physiological solution in a circulating cold-water bath. The mesenteric arcade was pinned out and 1A arterioles were identified under a microscope, the perivascular adipose tissue was removed and arteries were carefully dissected and cannulated on glass micropipettes within a pressure myograph (DMT Inc, Atlanta, GA, USA). Cannulated microvessels were then gradually pressurized to 68 cm H_2_O. Following 20 minutes of acclimatization to pressure, vessels were preconstricted with phenylephrine to at least 20% of starting lumen diameter. EDD was evaluated to increasing concentrations of acetylcholine (ACh, 10^−9^ to 10^−4^ M) in the presence and absence of the nitric oxide synthase inhibitor L-NAME (0.1 mM, 30 min) or following incubation with the superoxide scavenger TEMPOL (1 mM, 60 min). Endothelium-independent vasodilation was evaluated to increasing concentrations of sodium nitroprusside (SNP, 10^−10^ to 10^−4^ M). Vasodilation data are presented as the percentage of possible dilation after preconstriction to phenylephrine and vessel diameters were determined using MyoView software (DMT Inc., Atlanta, GA, USA). The half maximal effective concentration (EC_50_) was calculated using GraphPad Prism 9.4.0.

### Electron paramagnetic resonance

Carotid arteries were excised, cleaned of perivascular tissues, and 2 mm segments were cut from just below the bifurcation. Krebs/HEPEs buffer (KHB) was deoxygenated for 10 minutes with gaseous nitrogen and kept on ice. 0.002 g CMH was added to 1 mL KHB. Carotid artery segments were placed in 180 μL of KHB containing CMH and placed at 37ºC for 1 hour and then flash frozen in liquid nitrogen until analysis. Samples were analyzed using an MS300 X-band EPR Spectrometer (Magnettech, Berlin, Germany) and the following settings: microwave frequency 9.83 GHz, centerfield 3480 G, sweep 80 G, modulation amplitude 3.3 G, microwave power 40 mW, microwave attenuation 7, and receiver gain 30. Six total sweeps were conducted lasting 8.7s per sweep. The running average of the six sweeps was collected and the double integration (area under and over the baseline) of the triplet was used to display the magnitude of the signal. The magnitude of this signal directly relates to the amount of superoxide that has been trapped by the CMH. All values were normalized to old ATM +/+ superoxide production.

### Mesenteric microvascular density and endothelial glycocalyx measurements

Mesenteric perfused microvascular density, and perfused boundary region – a measure of glycocalyx thickness, were measured using intravital microscopy and an automated capture an analysis system as described previously [3]. Briefly, mice were anesthetized under 2% isoflurane on a heating pad set to 37ºC, and a small incision was made in the lower abdomen. The mesenteric arcade was gently moved from the abdominal cavity into a petri dish containing 0.9% isotonic saline solution warmed to 37ºC. To ensure similar measurement sites for all animals, the cecum was identified, and the intravital microscope was used to collect 5–10 videos per animal.

### Aortic pulse wave velocity

Mice were anesthetized under 2% isoflurane and body temperature was maintained using a heating pad set to 37ºC. 20-MHz Doppler probes (Indus Instruments, Webster, TX, USA) were placed on the aortic arch and the abdominal aorta, and WinDAQ Pro + software (DataQ Instruments, Akron, OH, USA) was used to record pulse waveforms. The distance between the two doppler probes was measured using a scientific caliper, and WinDAQ Waveform Browser (DataQ Instruments) was used to determine the transit time of pulse waveform between the doppler probes, using the foot-to-foot method as described previously [19, 21]. Distance between the sites divided by the transit time was calculated to determine PWV. 30 individual waveforms were analyzed per mouse.

### mRNA expression

The abdominal aorta was harvested, cleaned of perivascular adipose tissue, flash frozen in liquid nitrogen, and stored at −80ºC. RNA was extracted using an QIAgen RNEASY kit as per manufacturer guidelines, and cDNA was synthesized using QuantiTect Reverse Transcription Kit (Qiagen, Germantown, MD, USA) according to the manufacturer’s protocol. Quantitative PCR was performed using SsoFast EvaGreen Supermixes (Bio-Rad, Hercules, CA, USA) and Bio-Rad CFX™ Real Time system. The 2^−ΔΔCt^ was used to quantify relative gene expression. Primer sequences are provided in Supplementary Table 1.

### Statistical analysis

Statistical analysis was performed using GraphPad Prism 9.4.0. Group differences were determined by unpaired two-tailed *t*-tests, one-way ANOVA, or repeated measures two-way ANOVA with LSD post-hoc tests. Statistical significance was set at *p* < 0.05 and data are presented as mean ± SEM.

### Data availability statement

The data that support the findings of this study are available from the corresponding author upon reasonable request.

## RESULTS

**Figure 1:**
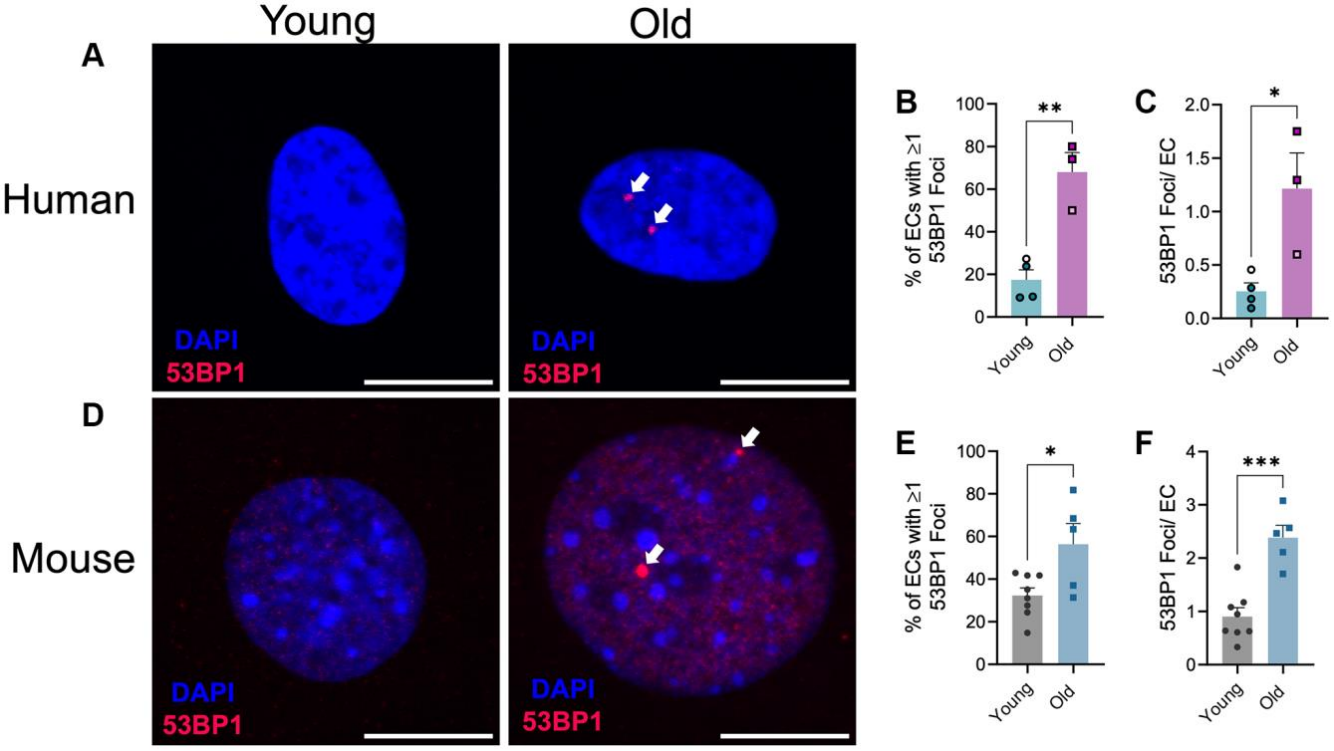
**Effect of aging on endothelial cell DNA damage.** (**A**) Representative images of immunofluorescence for the DNA damage marker 53BP1 on human microvascular lung endothelial cells from young (29 ± 1 year old, female) and old (67 ± 1 year old, female) donors. *N* = 3–4 experimental replicates from 2 young and 2 old donors. (**B**) Percentage of endothelial cells (EC) containing one or more 53BP1 foci. (**C**) Number of 53BP1 foci per endothelial cell. Experimental replicates on cells from the same donors are denoted by individual data points with like colors. (**D**) Representative images of immunofluorescence for the DNA damage marker 53BP1 on mouse microvascular lung endothelial cells from young (2.7 ± 0 mo old, male) and old (27 ± 0 mo old, male) mice. *N* = 5–8 experimental replicates from 12 young and 12 old mice per group. (**E**) Percentage of endothelial cells containing one or more 53BP1 foci. (**F**) Number of 53BP1 foci per endothelial cell. Individual data points with black borders denote females. Individual data points matching group colors denote males. ^*^p < 0.05, ^**^p < 0.01, ^***^p < 0.001. Scale bars are 10 μm.

We recently demonstrated that advanced age results in increases in DNA damage in endothelial cells and vascular smooth muscle cells within large arteries, namely from the aorta [17]. To determine if advanced age similarly results in microvascular DNA damage, we performed immunofluorescence for the DNA damage marker 53BP1, which plays a key role in double-strand DNA break repair [22], in lung microvascular endothelial cells from young and old human donors and young and old mice. Similar to large arteries, aging increased the percentage of endothelial cells containing one or more 53BP1 foci (Figure 1A, 1B, *p* < 0.05) and the number of 53BP1 foci per endothelial cell (Figure 1A, 1C, *p* < 0.001) in human lung microvascular endothelial cells. Likewise, in mouse lung endothelial cells, aging increased the percentage of endothelial cells containing one or more 53BP1 foci (Figure 1D, 1E, *p* < 0.05) and the number of 53BP1 foci per endothelial cell (Figure 1D, 1F, *p* < 0.001). Taken together these data provide direct evidence of the increased abundance of DNA damage within the vasculature in advanced age.

**Figure 2:**
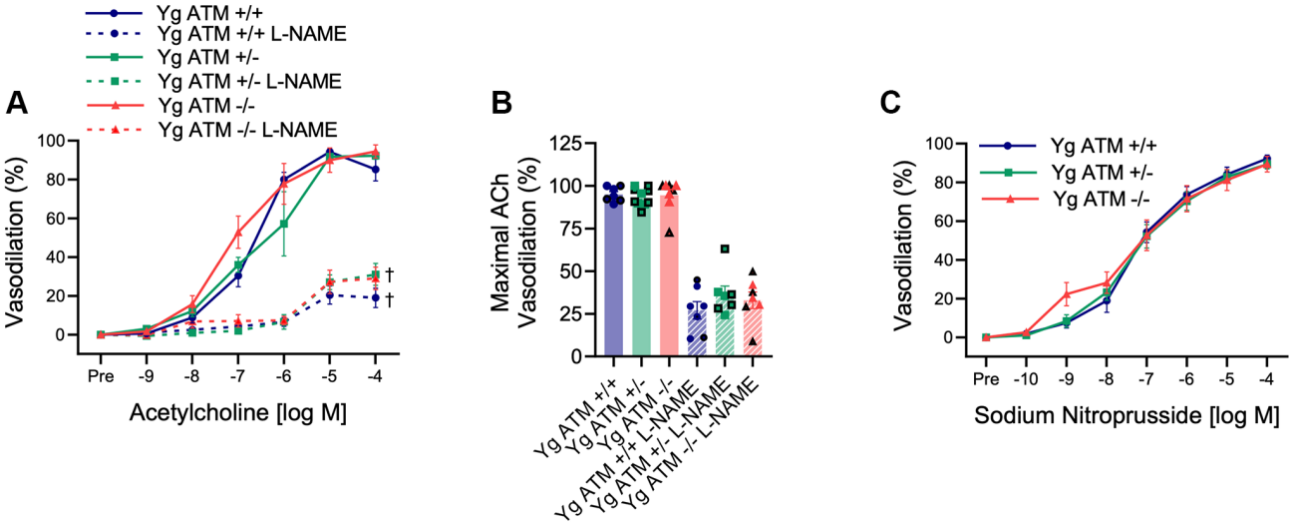
**Impact of reduced double-strand DNA break repair on endothelium-dependent and independent vasodilation.** (**A**) Mesenteric artery dose-response curves to increasing doses of the endothelium-dependent vasodilator acetylcholine in the absence and presence of the nitric oxide synthase inhibitor L-NAME. *N* = 7–11 per group. (**B**) Maximal acetylcholine (ACh) vasodilation in mesenteric arteries in the absence and presence of L-NAME. *N* = 7–11 per group. (**C**) Mesenteric artery dose-response curves to increasing doses of the endothelium-independent vasodilator sodium nitroprusside. Yg = young. *N* = 8–10 per group. Individual data points with black borders denote female mice. Individual data points matching group colors denote male mice. ^†^*p* < 0.05 vs. Acetylcholine dose-response curve in the absence of L-NAME from the same group.

Next, we sought to determine the vascular consequences of increases in DNA damage, and in particular double-strand DNA breaks which are regarded as the most deleterious form of DNA damage. To evaluate this, we examined young (Yg, ~5 mo), mice that were wildtype (ATM +/+), heterozygous (ATM +/−), or knockout (ATM −/−) for the double-strand DNA break repair protein ATM kinase. Mouse characteristics are displayed in Table 1. We began by evaluating endothelial function in mesenteric arteries. Interestingly, EDD to acetylcholine (ACh) was unaffected in young ATM +/− or ATM −/− mice compared to ATM +/+ littermate controls (Figure 2A, 2B, *p* > 0.05). Furthermore, nitric oxide (NO) bioavailability was unaffected in young ATM +/− or ATM −/− mice, evidenced by no difference in vasodilation to ACh in the presence of the NO synthase inhibitor L-NAME between genotypes (Figure 2A, 2B, *p* > 0.05). Furthermore, vascular smooth muscle cell responsiveness to NO was not different between groups (Figure 2C, *p* > 0.05). Taken together, these data indicate that in young mice, genetic reduction of the double-strand DNA break repair protein ATM kinase does not impact endothelium-dependent or independent vasodilation.

Because young mice display low levels of DNA damage, whereas aging increases DNA damage in both large arteries [17] and the microvasculature (Figure 1), we rationalized that young mice may not have experienced enough genotoxic stress for vascular function to be impacted by reduced ATM kinase. Therefore, to examine the impact of increased DNA damage, we aged ATM +/+ and ATM +/− mice to ~18 mo. This age was chosen because it is prior to the time in which ATM +/− mice die (~19 mo). ATM −/− mice were not included in the aging studies because these mice die at ~6 mo, typically from lymphoma [23]. The characteristics of aged mice are displayed in Table 1. Sex differences were detected in body weight between male and female old ATM +/+ mice (*p* < 0.05), and several tissue weights within genotypes. However, when normalized to body weight, only spleen weight was greater in old ATM +/+ female mice versus old ATM +/+ male mice (*p* < 0.05), and liver weight was greater in male versus female old ATM +/− mice (*p* < 0.05). Because there were no other sex differences for body weight, tissue weight, or cellular or physiological measures, male and female mice were grouped together for the remaining outcomes.

**Figure 3:**
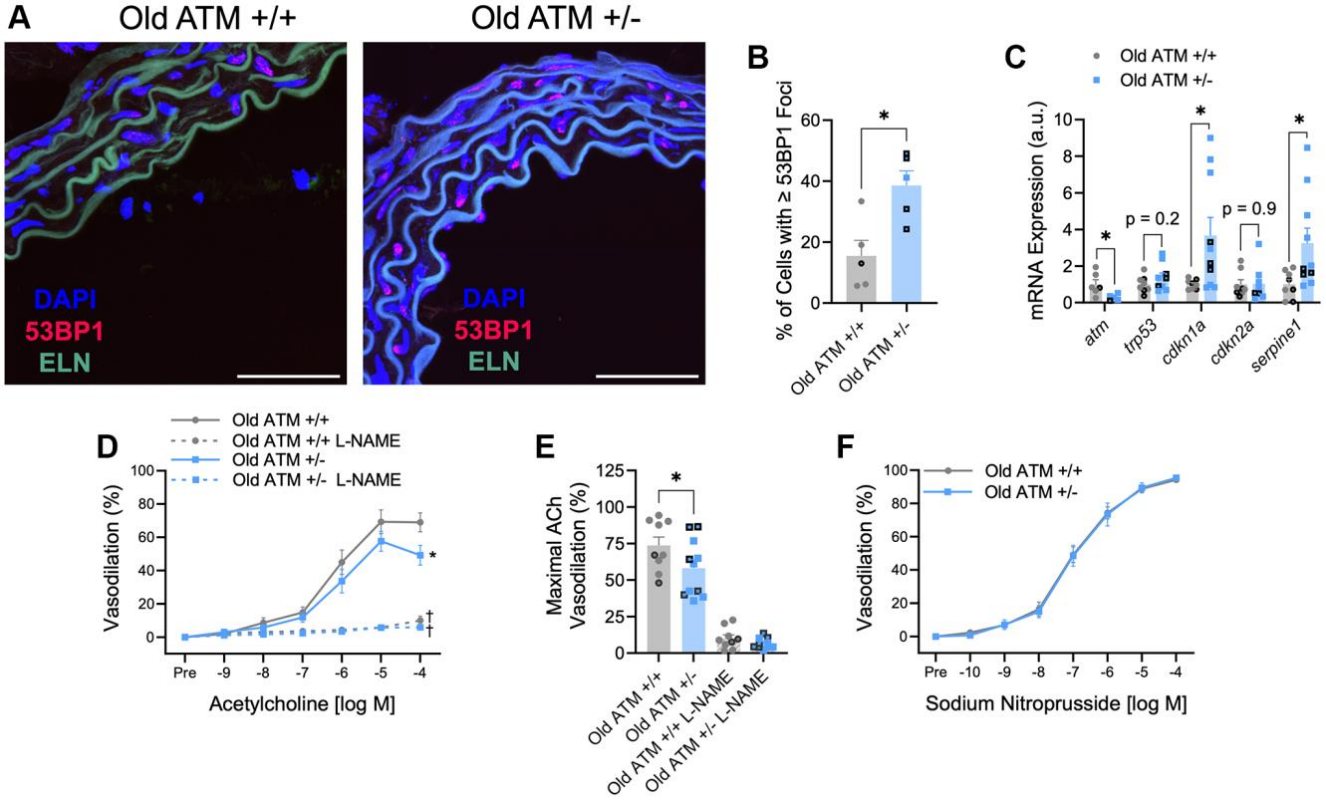
**Impact of aging and reduced double-strand DNA break repair on arterial DNA damage, senescence, and endothelium-dependent and -independent vasodilation.** (**A**) Representative images of immunofluorescence for the DNA damage marker 53BP1 performed in aortic segments of Old ATM +/+ and Old ATM+/− mice. Green is elastin (ELN) autofluorescence from tunica media. *N* = 4–6 per group. (**B**) Percentage of aortic cells containing one or more 53BP1 foci. (**C**) Aortic mRNA expression of atm and senescence-related genes. *N* = 5–10 per group. (**D**) Mesenteric artery dose-response curves to increasing doses of the endothelium-dependent vasodilator acetylcholine in the absence and presence of the nitric oxide synthase inhibitor L-NAME. *N* = 8–14 per group. (**E**) Maximal acetylcholine (ACh) vasodilation in mesenteric arteries in the absence and presence of L-NAME. *N* = 8–14 per group. (**F**) Mesenteric artery dose-response curves to increasing doses of the endothelium-independent vasodilator sodium nitroprusside. *N* = 14–16 per group. Individual data points with black borders denote female mice. Individual data points matching group colors denote male mice. ^*^p < 0.05 vs. Old ATM +/+ Acetylcholine dose-response curve in the absence of L-NAME. ^†^*p* < 0.05 vs. Acetylcholine dose-response curve in the absence of L-NAME from the same group. Scale bars are 50 μm.

Compared to old ATM +/+ mice, old ATM +/− mice displayed increased arterial DNA damage evaluated as the percentage of cells containing 53BP1 foci, as well as increased arterial senescence gene expression (Figure 3A–3C, *p* < 0.05). Aging reduced maximal EDD in ATM +/+ mice by ~23% (Figure 2B and 3E, Supplementary Figure 1B, *p* < 0.01). EDD was reduced to a greater extent, ~38%, in Old ATM +/− mice compared to their Yg ATM +/−counterparts (Figure 2B and 3E, Supplementary Figure 1B, *p* < 0.0001). Furthermore, vasodilation to ACh was reduced in old ATM +/− compared to old ATM +/+ mice (Figure 3D, 3E, Supplementary Figure 1A, 1B, *p* < 0.05). However, vasodilation to ACh in the presence of L-NAME was not different between old ATM +/− and old ATM +/+ mice (Figure 3D, 3E, *p* > 0.05) suggesting that reductions in EDD resulted from reductions in the bioavailability of NO. Vascular smooth muscle cell responsiveness to NO remained intact in old ATM +/+ and old ATM +/− mice evidenced by similar vasodilatory responses to sodium nitroprusside (Figure 3F, *p* > 0.05). These data demonstrate that aging, which increases arterial DNA damage, in conjunction with a genetic reduction in the double-strand DNA break repair protein ATM kinase, accelerates impairments in EDD.

**Figure 4:**
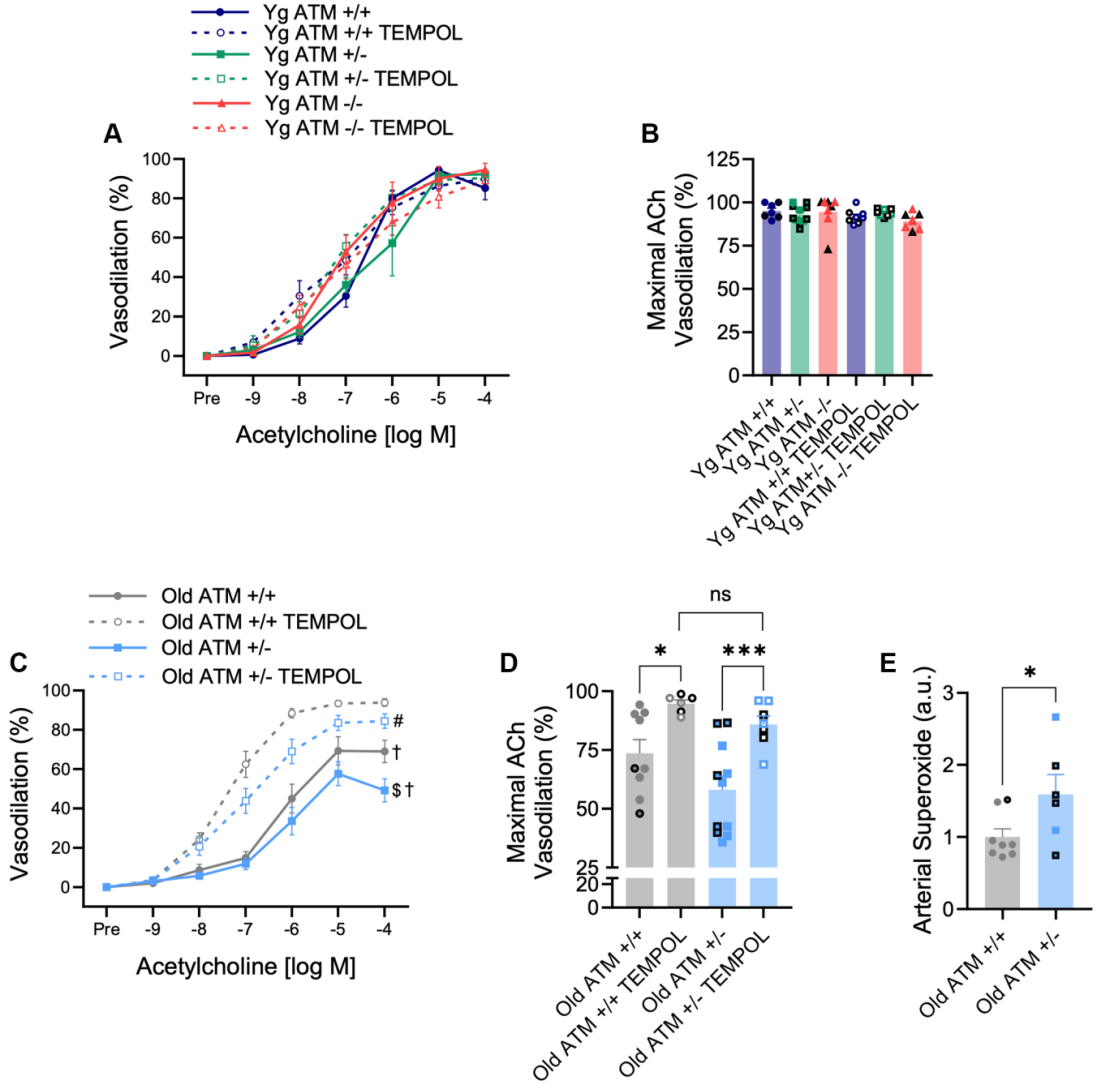
**Effect of aging and reduced DNA double-strand break repair on oxidative stress-mediated suppression of vasodilation.** (**A**) mesenteric artery dose-response curves to increasing doses of the endothelium-dependent vasodilator acetylcholine in the absence and presence of the superoxide scavenger TEMPOL. *N* = 7–11 per group. (**B**) Maximal acetylcholine (ACh) vasodilation in mesenteric arteries in the absence and presence of TEMPOL. *N* = 7–11 per group. (**C**) Mesenteric artery dose-response curves to increasing doses of the endothelium-dependent vasodilator acetylcholine in the absence and presence of the superoxide scavenger TEMPOL. *N* = 6–11 per group. (**D**) Maximal acetylcholine (ACh) vasodilation in mesenteric arteries in the absence and presence of TEMPOL. *N* = 6–11 per group. (**E**) Carotid artery superoxide production measured via electron paramagnetic resonance spectrometry. *N* = 6–8 per group. Individual data points with black borders denote female mice. Individual data points matching group colors denote male mice. ^$^*p* < 0.05 vs. Old ATM +/+ Acetylcholine dose-response curve in the absence of TEMPOL, ^†^*p* < 0.05 vs. Acetylcholine dose-response curve in the presence of TEMPOL from the same group, ^#^*p* < 0.05 vs. Old ATM+/+ Acetylcholine dose-response curve in the presence of TEMPOL, ^*^p < 0.05, ^***^p < 0.001.

In advanced age, the bioavailability of NO is quenched by increases in oxidative stress. To examine if reductions in EDD occur via similar mechanisms in mice with reduced double-strand DNA break repair, we repeated dose responses to ACh following preincubation of arteries with the superoxide scavenger TEMPOL. As expected, vasodilation in the presence of TEMPOL did not impact vasodilation in young mice, independent of ATM (Figure 4A, 4B, *p* > 0.05). However, TEMPOL increased vasodilation in both old ATM +/+ and old ATM +/− mice (Figure 4C, 4D, *p* < 0.05). Maximal vasodilation in the presence of TEMPOL was not different between old ATM +/+ and old ATM +/− mice (Figure 4D, *p* > 0.05), suggesting that increases in oxidative stress in old ATM +/− compared to old ATM +/+ mice were responsible for impairments in EDD. None of the changes seen in vasodilation were caused by altered sensitivity to ACh as the half maximal effective concentration (EC_50_) of ACh did not vary between groups (Table 2, *p* > 0.05). To confirm that old ATM +/− mice have increased arterial oxidative stress compared to old ATM +/+ mice, we isolated carotid artery segments and performed electron paramagnetic resonance spectrometry to measure superoxide production using the spin trap CMH. Congruent with the vasodilation responses seen in the presence of TEMPOL, arteries from old ATM +/− mice produced more superoxide than arteries from old ATM +/+ mice (Figure 4E, *p* < 0.05). Collectively, these data demonstrate that advancing age combined with reduced double-strand DNA break repair increases oxidative stress that quenches NO resulting in impairments in EDD.

**Table 2:** EC50 of mesenteric artery dose-response curves.

**Genotype**	**Yg ATM +/+**	**Yg ATM +/−**	**Yg ATM −/−**	**Old ATM +/+**	**Old ATM +/−**
EC_50_, log *M*					
ACh	−6.7 ± 0.1	−6.5 ± 0.2	−6.8 ± 0.3	−6.4 ± 0.3	−6.2 ± 0.1
ACh + L-NAME	−6.5 ± 0.5	−5.5 ± 0.3	−5.8 ± 0.5	−6.0 ± 0.5	−6.4 ± 0.4
SNP	−7.2 ± 0.2	−7.3 ± 0.2	−7.1 ± 0.4	−7.0 ± 0.1	−6.9 ± 0.2
ACh + TEMPOL	−7.4 ± 0.3	−7.3 ± 0.2	−7.8 ± 0.2	−7.3 ± 0.1	−6.8 ± 0.3

Data are mean ± SEM.

**Figure 5:**
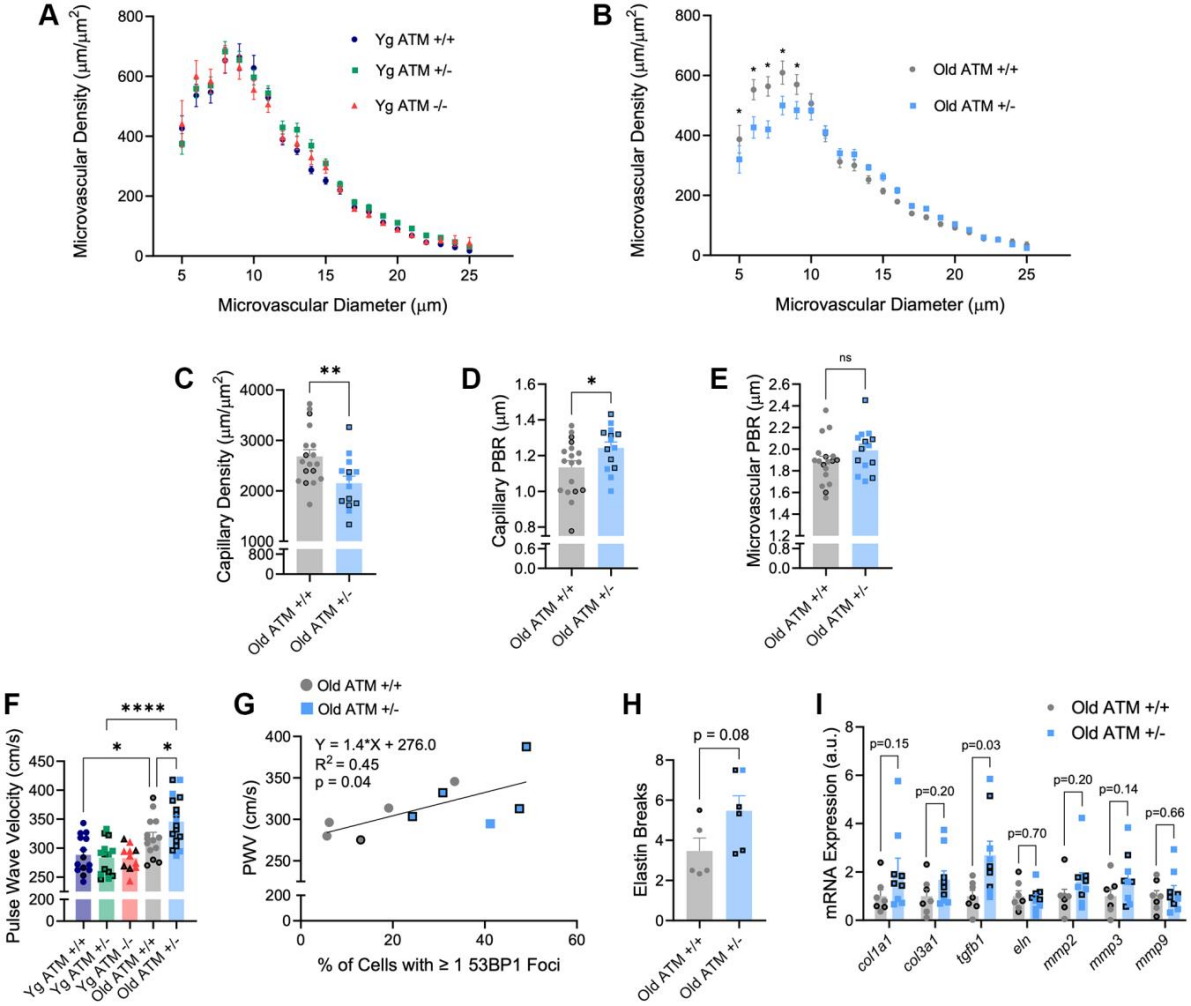
**Effect of aging and reduced DNA double-strand break repair on micro- and macro-vascular properties.** (**A**) Perfused microvascular density in mesenteric microcirculation. *N* = 10–15 per group. (**B**) Perfused microvascular density in mesenteric microcirculation. *N* = 14–18 per group. (**C**) Density of perfused capillaries between 5–9 μm in size from mesenteric microcirculation. *N* = 14–18 per group. (**D**) Perfused boundary region in capillaries between 5–9 μm in size in mesenteric microcirculation. *N* = 14–18 per group. (**E**) Perfused boundary region in all microvessels between 10–25 μm in size in mesenteric microcirculation. *N* = 14–18 per group. (**F**) Aortic stiffness assessed via pulse wave velocity. *N* = 11–17 per group. (**G**) Positive relation between PWV and percentage of aortic cells containing one or more 53BP1 foci. *N* = 5 per group. (**H**) Aortic elastin breaks. *N* = 5–6 per group. (**I**) Aortic mRNA expression of extracellular matrix and arterial stiffness-related genes. *N* = 7–9. Individual data points with black borders denote female mice. Individual data points matching group colors denote male mice. ^*^p < 0.05, ^**^p < 0.01, ^****^p < 0.0001.

In addition to impairments in endothelial function, aging is associated with alterations in microvascular properties including reductions in microvascular density and glycocalyx thickness. To examine the impact of reduced ATM kinase on microvascular density, we utilized intravital microscopy paired with an automated capture and analysis system. Similar to endothelial function, young ATM +/− or ATM −/− mice did not display overt alterations in perfused vascular density in the mesenteric microcirculation compared to ATM +/+ controls (Figure 5A, *p* > 0.05). In comparison to young ATM +/+ mice, old ATM +/+ mice did not have a reduction in capillary density, (young: 2826.7 ± 157.4 vs. old: 2682.6 ± 131.2, *p* > 0.05), but had reductions in the density of microvessels between 10–25 μm in size (young: 3370.7 ± 149.3 vs. old: 2899.8 ± 130.8, *p* < 0.05). In old ATM +/− mice, both capillary density and microvessels between 10–25 μm in size were reduced compared to young ATM +/− mice (capillary density: young: 18250 ± 115.3 vs. 2151.8 ± 138.0, *p* < 0.001. 10–25 μm microvessels: young: 3864.9 ± 133.1 vs. old: 3153.7 ± 100.0, *p* < 0.0001). Old ATM +/− mice had reduced microvascular density compared to old ATM +/+ mice (Figure 5B, *p* < 0.05), which was driven largely by a reduction in the number of perfused capillaries (Figure 5C, *p* < 0.01. Examination of the perfused boundary region (PBR), a marker of glycocalyx thickness, revealed that old ATM +/− mice have greater PBR, indicative of a diminished endothelial glycocalyx in capillaries between 5–9 μm (Figure 5D, *p* < 0.05) but not microvessels between 10–25 μm within the mesenteric circulation (Figure 5E, *p* > 0.05). Taken together, these data demonstrate that a reduction of the double-strand DNA break repair protein ATM kinase in advancing age exacerbates microvascular dysfunction.

With advancing age, stiffening of large arteries as measured by PWV is a strong independent predictor of CVD. Based on the importance of PWV, we sought to determine the impact of genetic reductions in ATM on PWV. In young mice, PWV did not differ between ATM genotypes (Figure 5F, *p* > 0.05), though heart rate was reduced in young ATM −/− mice compared to young ATM +/+, whereas heart rate in young ATM +/− mice was not different (Yg ATM +/+: 466 ± 11 bpm; Yg ATM +/−: 441 ± 13; Yg ATM −/−: 405 ± 11). Similar to prior data published by our group, PWV was significantly increased in ATM +/+ mice at ~18 mo of age compared to young ATM +/+ mice [21]. Old ATM +/− mice displayed elevated PWV compared to ATM +/+ mice at the same age (Figure 5F, *p* < 0.05) and PWV values from old ATM +/− mice resembled values seen in ~24 mo old mice [21, 24]. In old mice, changes in PWV occurred independently of differences in heart rate (old ATM+/+: 435 ± 11 bpm; old ATM +/− 460 ± 14 bpm, *p* > 0.05). PWV was positively correlated with the percentage of aortic cells containing one or more 53BP1 foci (Figure 5G, R^2^ = 0.45, *p* < 0.05). Old ATM +/− mice tended to have a greater number of elastin breaks in aortic sections compared to old ATM +/+ mice (Figure 5H, *p* = 0.08). Moreover, aortic gene expression of transforming growth factor beta 1 (*tgfb1*) was elevated in old ATM +/− mice compared to ATM +/+ mice, whereas other genes associated with arterial stiffening tended to be elevated or were not different between groups (Figure 5I). These data suggest that the reduction of double-strand break repair protein ATM kinase accelerates increases in arterial stiffness, an important hallmark of vascular aging.

## DISCUSSION

The key novel findings of the present study are as follows: First, aging increases DNA damage in microvascular endothelial cells. Second, reducing DNA damage repair capacity by genetic reduction of ATM kinase does not impact EDD in young mice; however, in comparison to ATM +/+ mice, aging ATM +/− mice to ~18 mo increases arterial DNA damage, senescence signaling, and reduces EDD due to elevated production of arterial oxidative stress. Third, reductions in ATM do not impact microvascular density or glycocalyx thickness in young mice, whereas ATM heterozygosity in aged mice results in a greater loss of microvascular density and increased penetrability of the glycocalyx than is seen in ATM +/+ mice. Finally, aging ATM +/− mice accelerates age-related changes in arterial stiffness. Collectively, these data highlight the accumulation of DNA damage in arteries in advanced age as a potential mechanism responsible for arterial dysfunction.

Evidence demonstrating the abundance of DNA damage in the vasculature with healthy aging is limited and has only been examined in large arteries [17]. Accordingly, we first sought to determine the amount of DNA damage in microvascular lung endothelial cells. Lung microvascular endothelial cells were utilized because human endothelial cells from young and old donors are difficult to obtain, and we were able to identify lung endothelial cells from a commercial vendor. To determine if these findings in humans translated to mice, and because the lung is highly vascularized increasing feasibility, we also evaluated lung endothelial cells in mice. Similar to endothelial cells and vascular smooth muscle cells of the mouse aorta [17], aging increased the percentage of lung endothelial cells containing 53BP1 foci, as well as the number of 53BP1 foci per cell in both humans and mice. These findings are important because they suggest that DNA damage in the vasculature occurs within multiple vascular beds and cell types.

DNA damage in vascular cells with advancing age is probable and particularly consequential for several reasons. First, through exposure to the circulation, cells of the vasculature interact with higher partial pressures of oxygen compared to other tissues, as well as pulsatile and oscillatory blood flow, circulating immune cells, and metabolites, all of which may induce DNA damage [25]. Additionally, because every organ requires a vascular network, cells within the vasculature may be exposed to DNA-damaging reactive oxygen species, inflammation, and metabolites derived from dysfunctional tissues. In line with this logic, vascular dysfunction due to DNA damage could have systemic consequences by impairing tissue function in a wide variety of organs [25, 26]. As vascular cells are generally long-lived cell types [14], DNA damage that results in mutations may have long-term effects. This idea is supported by the fact that treating cancer patients with genotoxic chemotherapy or radiation increases the risk of age-related CVDs many years after treatment [27–29]. The consequences of DNA damage in the vasculature are further exemplified by genetic defects in DNA damage repair and maintenance proteins which result in accelerated aging syndromes that often lead to premature CVD and death [9, 30]. Cumulatively, these findings highlight the importance of identifying the incidence of DNA damage in the vasculature in advanced age.

To elucidate the physiological consequences of DNA damage in the vasculature, we began by evaluating EDD in mice with heterozygous or homozygous knockout of ATM kinase, a protein that orchestrates the double-strand DNA break response [13]. Surprisingly, genetic alteration of ATM did not impact EDD in young mice. This finding led us to rationalize that because young animals display low levels of arterial DNA damage, perhaps more time would be required to accumulate damage and thus evaluate the impact of reduced double-strand DNA break repair capacity. Mice lacking both copies of ATM die at 4–6 mo of age. Therefore, to examine the impact of elevated DNA damage, we aged ATM +/+ and +/− mice to ~18 mo, prior to the age at which old ATM +/− mice die. At ~18 mo, old ATM +/− mice displayed increased arterial DNA damage compared to ATM +/+ mice. Furthermore, markers of senescence, including the cyclin-dependent kinase inhibitor *cdkn1a* (also known as p21) as well as *serpine1* (also known as pai-1), one of the most common and abundant factors produced by senescent cells [31, 32], were both elevated in old ATM +/− mice. Functionally, old ATM +/− mice had impaired EDD in mesenteric arterioles resulting from reduced NO bioavailability due to increased oxidative stress. Other measures of vascular aging demonstrated similar trends, as young mice displayed no overt alterations in microvascular density, glycocalyx thickness, or arterial stiffness. In contrast, old ATM +/− mice exhibited an accelerated vascular aging phenotype characterized by reductions in perfused capillary density and microvascular glycocalyx thickness, and increased large artery stiffness. Alterations in endothelial function, microvascular properties, and arterial stiffness precede and contribute to CVD in advanced age [4–6]. Interestingly, the magnitude of increases in both DNA damage and senescence signaling appear greater than the alterations in vascular function. Several lines of logic could explain this finding. First, DNA damage may result in a few mutations with functional consequences as a result of the degeneracy of the genetic code, or secondly, a large senescence burden may be required to impair endothelial function. Finally, it is also possible that the consequences of age-related increases in DNA damage in the vasculature act synergistically with other hallmarks of aging to drive impairments in function. To understand which contributes most to the phenotypes observed in the present study, future studies could employ genomic and RNA sequencing. Nonetheless, the findings of the present study implicate the accumulation of DNA damage as a putative contributing mechanism responsible for age-related vascular dysfunction in the absence of overt CVD, which is supported by studies utilizing other models of DNA damage repair deficiency and deprotection [20, 33, 34].

Beyond age-related vascular dysfunction, studies have found that DNA damage contributes to atherosclerotic CVD. For example, several groups have demonstrated that ATM heterozygosity in mice on an Apo E null background have increased atherosclerotic plaque burden [35–37]. These data are supported by human studies, in which individuals with a single mutant allele of ATM, which comprise approximately one percent of the population, are at greater risk of developing atherosclerotic CVD [38–41]. Because the vascular aging phenotypes assessed in the present study precede atherosclerosis, our findings suggest a model where aging results in the accumulation of DNA damage in arterial cells that impairs vascular function which leads to the development of CVD. Outside of the vasculature, a possible way that DNA damage could contribute to the phenotypes observed here, as well as atherosclerosis, is that mutations could drive clonal expansion of inflammatory immune cells, known as clonal hematopoiesis of indeterminant potential (CHIP). ATM kinase is one of the most commonly mutated proteins associated with the incidence of CHIP [42, 43], CHIP is known to contribute to atherosclerosis [44], and inflammatory immune cells contribute to vascular aging phenotypes [24]. Based on this understanding, studies of vascular function in humans harboring mutations in one allele of ATM prior to the development of atherosclerosis would be of great interest.

### Conclusions, limitations, and future directions

The findings of the present study support the conclusion that DNA damage contributes to vascular aging, however, future studies on this topic are warranted. In the present study, we examined 53BP1 as a marker of DNA damage, as ATM kinase is the major kinase involved in the phosphorylation of H2AX, forming γ-H2AX, another commonly used marker of DNA damage [45]. Therefore, to examine DNA damage independent of ATM gene status, we evaluated 53BP1. Future studies that provide a more detailed analysis of DNA damage in the vasculature by examining other markers (e.g., γ-H2AX), types of DNA lesions, duration of damage and repair capacity/fidelity, would be of great interest and importance. A major finding of the present study is that in the context of aging, reductions in ATM kinase impair endothelial function in mesenteric arterioles through increases in oxidative stress. Moreover, microvascular density, and glycocalyx properties were reduced, which collectively highlights the microvascular consequences of increased DNA damage with aging. However, whether these changes are seen in microvasculature from other tissues (e.g., skeletal muscle) would also be of interest. In relation to large arteries, aortic stiffness was increased, which was associated with the abundance of DNA damage. Because blood pressure can influence arterial stiffness, additional studies examining whether blood pressure is altered by increases in DNA damage would be of interest and importance. Finally, a thorough analysis of the effects of biological sex on these outcomes is warranted.

In conclusion, the findings of the present study provide important evidence of the incidence of arterial DNA damage in advanced age. Furthermore, these findings support the notion that the accumulation of DNA damage results in functional consequences including impairments in endothelial function and microvascular properties, as well as increased arterial stiffness. Cumulatively, this suggests a model where the accumulation of DNA damage with aging impairs vascular function ultimately resulting in CVD.

## Supplementary Materials

Supplementary Figure 1

Supplementary Table 1
