# Supplementary material for: Reduction of double-strand DNA break repair exacerbates vascular aging
Source: Aging (Albany NY). 2023 Oct 2;15(19):9913–47. doi: 10.18632/aging.205066 (PMC10599741; doi:10.18632/aging.205066)
Supplement: Supplementary Figure 1 [file aging-15-205066-s001.pdf]

SUPPLEMENTARY FIGURE

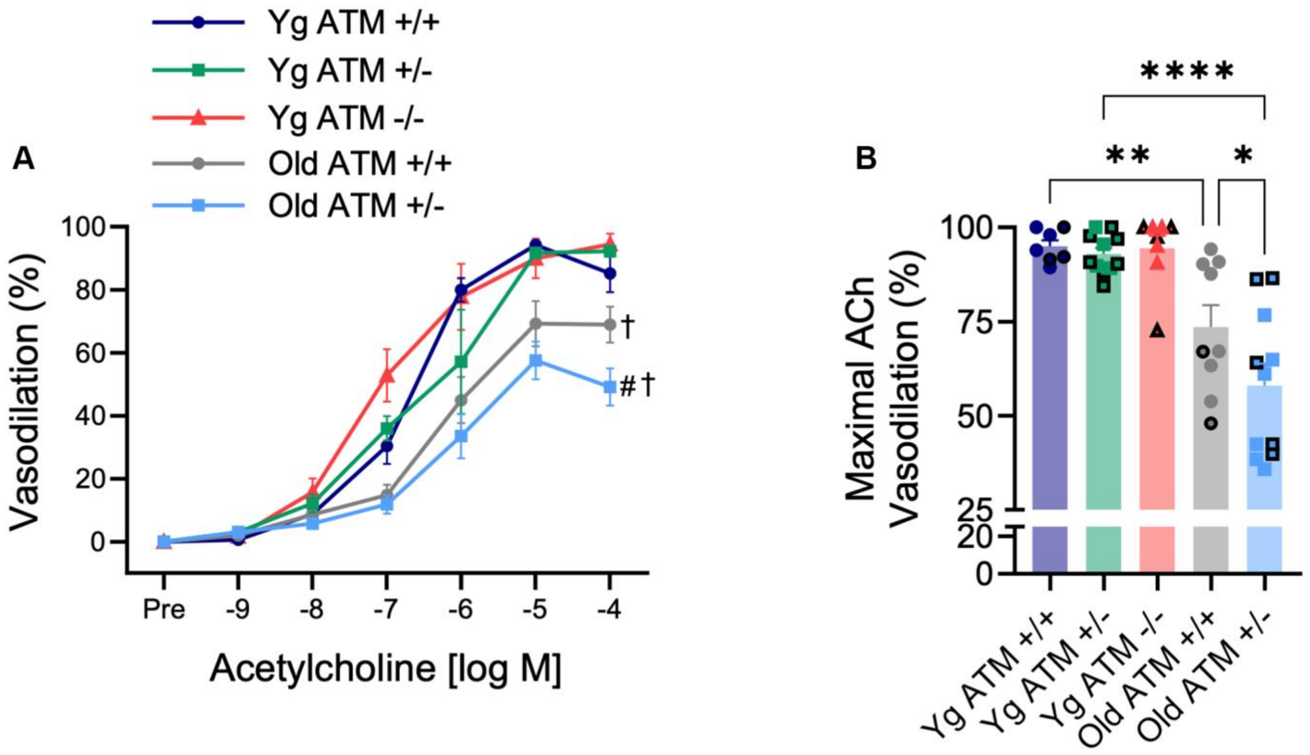

Supplementary Figure 1. Impact of aging and reduced double-strand DNA break repair on endothelium-dependent vasodilation. (**A**) Mesenteric artery dose-response curves to increasing doses of the endothelium-dependent vasodilator acetylcholine. *N* = 7–11 per group. (**B**) Maximal acetylcholine (ACh) vasodilation in mesenteric arteries. *N* = 7–11 per group. Individual data points with black borders denote female mice. Individual data points matching group colors denote male mice. $^{\#}p < 0.05$ vs. Old ATM +/+ Acetylcholine dose-response curve in the absence of L-NAME. $^{\dagger}p < 0.05$ vs. Yg mouse Acetylcholine dose-response curve from the same genotype. $^{*}p <$ 0.05, $^{**}p < 0.01$, $^{****}p < 0.0001$.
