# Supplementary material for: Reduction of double-strand DNA break repair exacerbates vascular aging
Source: Aging (Albany NY). 2023 Oct 2;15(19):9913–47. doi: 10.18632/aging.205066 (PMC10599741; doi:10.18632/aging.205066)
Supplement: Supplementary Table 1 [file aging-15-205066-s002.pdf]

## SUPPLEMENTARY TABLE

**Supplementary Table 1. Primer sequences for RT-qPCR.**

| Genes | Forward | Reverse |
|---|---|---|
| *18s* | 5′-TAGAGGGACAAGTGGCGTTC-3′ | 5′-CGCTGAGCCAGTCAGTGT-3′ |
| *atm* | 5′-AGGCTGTTGGAAGCTGCTGG-3′ | 5′-CTAGTAATGGGTTGTAACATC-3′ |
| *trp53* | 5′-CTCTCCCCCGCAAAAGAAAAA-3′ | 5′-CGGAACATCTCGAAGCGTTTA-3′ |
| *cdkn2a* | 5′-CGCAGGTTCTTGGTCACTGT-3′ | 5′-TGTTCACGAAAGCCAGAGCG-3′ |
| *cdkn1a* | 5′-CCTGGTGATGTCCGACCTG-3′ | 5′-CCATGAGCGCATCGCAATC-3′ |
| *serpine1* | 5′-GACACCCTCAGCATGTTCATC-3′ | 5′-AGGGTTGCACTAAACATGTCAG-3′ |
| *col1a1* | 5′-TAAGGGTCCCCAATGGTGAGA-3′ | 5′-GGGTCCCTCGACTCCTACAT-3′ |
| *col3a1* | 5′-GAGGGCCATAGCTGAACTGA-3′ | 5′-TGACTGTCCCACGTAAGCAC-3′ |
| *eln* | 5′-TCCATCCGCCCTGGTTAT-3′ | 5′-TGGCAGTCTGGTCCTCTAAAG-3′ |
| *tgfβ1* | 5′-CTCCCGTGGCTTCTAGTGC-3′ | 5′-GCCTTAGTTTGGACAGGATCTG-3′ |
| *mmp2* | 5′-CAAGTTCCCCGGCGATGTC-3′ | 5′-TTCTGGTCAAGGTCACCTGTC-3′ |
| *mmp3* | 5′-ACATGGAGACTTTGTCCCTTTTG-3′ | 5′-TTGGCTGAGTGGTAGAGTCCC-3′ |
| *mmp9* | 5′-CTGGACAGCCAGACACTAAAG-3′ | 5′-CTCGCGGCAAGTCTTCAGAG-3′ |
